# Erie County Medical Society

**Published:** 1865-06

**Authors:** 


Erie County Medical Society.—The Semi-Annual Meeting of Erie County Medical Society was held June 13th, and the following physicians were voted members upon compliance with the by-laws: Drs. Cole of Sardinia, Gleason, Little and Burger of Buffalo.

The meeting was quite fully attended. A large number of members from the towns were present, and the meeting was a spirited and attractive one. We learn that no address was delivered. Dr. Brown was appointed orator for the next meeting, and Dr. Johnson, substitute.

